# Investigating the Relationship Between Body Shape and Life History Traits in Toothed Whales: Can Body Shape Predict Fast-Slow Life Histories?

**DOI:** 10.1007/s11692-023-09605-4

**Published:** 2023-05-23

**Authors:** Steven H. Ferguson, Jeff W. Higdon, Chloe Schmidt, Corinne Pomerleau, Cory J. D. Matthews

**Affiliations:** 1 Fisheries & Oceans Canada, 501 University Crescent, Winnipeg, MB R3T 1M6 Canada; 2 Higdon Wildlife Consulting, 912 Ashburn Street, Winnipeg, MB R3G 3C9 Canada; 3 German Centre for Integrative Biodiversity Research (iDiv) Leipzig-Halle-Jena, Puschstraße 4, 04103 Leipzig, Germany; 4 National Defence, Government of Canada, Nanoose Bay, BC V9P 9J9 Canada

**Keywords:** Fast-slow continuum, Life history traits, Morphology, Odontocete, Predation selection pressure

## Abstract

A widespread pattern in vertebrate life-history evolution is for species to evolve towards either fast or slow life histories; however, the underlying causes of this pattern remain unclear. Toothed whales (Odontoceti) are a diverse group with a range of body sizes and life histories, making them an ideal model to investigate potential drivers of this dichotomy. Using ancestral reconstruction, we identified that certain groups of odontocetes evolved more-streamlined, presumably faster, body shapes around the same time that killer whales (*Orcinus orca*) evolved into whale predators approximately 1 Mya during the Pleistocene. This suggests that the evolution of a streamlined body shape may have been an adaptation to escape killer whale predation, leading to longer life-history events. To test this hypothesis, we performed a cluster analysis of odontocete whales and confirmed the dual pattern of life-history traits, with one group referred to as ‘reproducers’ characterized by early age of maturity, short gestation, short interbirth interval, and short lifespan, and the other group referred to as ‘bet-hedgers’ exhibiting the opposite pattern. However, we found that life history grouping was relatively unrelated to whale shape (i.e., more streamlined or less streamlined). Therefore, we incorporated principal component results into mixed effects models, and the model results indicated that body shape was positively related to neonate length (a measure of investment in progeny), but not significantly related to the temporal life-history traits. Thus, whale body shape is not a sufficient explanation for the evolution of fast-slow life histories in odontocete whales.

**Supplementary Information:**

The online version contains supplementary material available at 10.1007/s11692-023-09605-4.

## Introduction

Understanding the ecological factors that drive patterns of life‐history diversification is a central goal of evolutionary biology (Rolf, 1992; Stearns, 1976). Vertebrates often exhibit distinct evolutionary strategies, evolving towards either fast or slow life histories, even when controlling for body size (Dobson & Oli, 2007; Ferguson & Higdon, 2013; Read & Harvey, 1989; Ross, 1991). Lagomorphs, for example, exemplify a fast life history with short life spans, high reproductive rates, and low parental investment, while bats represent a slow mammalian life history characterized by long life spans, low reproductive rates, and high parental investment (Blueweiss et al., 1978; Dobson & Oli, 2008; Jones & MacLarnon, 2001). These life-history strategies are shaped by the costs and benefits associated with different ecological pressures, such as predation risk and foraging efficiency (Caddy, 2008; McNamara et al., 2008; Weimerskirch, 2001).

Morphological specialization in locomotion is influenced by an organism’s greater need to either search and capture prey to gain reproductive energy or evade predators to minimize mortality risk (Abrams, 2003; Huey & Pianka, 1981; Urban, 2007). In marine mammals, a streamlined body shape is a characteristic feature (Webb, 1984) that benefits swimming capabilities, as it reduces drag during swimming (Wassersug & Hoff, 1985), and thus should play a role in predator escape abilities (Dayton et al., 2005). Burst speed, a trait that is correlated with survivorship in a number of taxa, including fish, anurans, lizards, and mammals, is also influenced by body shape (Miles, 2004; O'Steen et al., 2002; Teplitsky et al., 2005; Wirsing, 2003). A streamlined body shape typically includes a rounded leading edge or head that tapers slowly to the tail (Videler, 1981), with a length/girth ratio that optimizes drag reduction and volume accommodation (Williams, 2018). Studies on a wide range of marine mammals have shown that many species conform to an ideal hydrodynamic range of around 4.5 for the length/girth ratio, indicating an optimized body shape for efficient swimming (Williams, 2018). Cetaceans, including odontocete whales, exhibit a range of body shapes with length/girth ratios from 3.0 to 8.0, reflecting different levels of streamlining that may be specialized for diving foraging efficiency or predator evasion (Fish, 1993; Kooyman, 1985).

Previous research has also suggested that life-history strategies in cetaceans (mysticetes & odontocetes) can be categorized into two groups: bet-hedgers, which exhibit reduced investment in progeny and long temporal life-history events, and reproducers, which exhibit greater investment in progeny and short timing of events (Ferguson & Higdon, 2013). However, while the temporal life-history traits align with the fast-slow continuum, these two groups do not conform perfectly to it. Possible explanations by the authors for this dichotomous pattern included differences in diet, environment, and predation, but body morphology has also been proposed as a contributing factor to life-history strategies (Gotthard & Nylin, 1995; Zhang, 2006). For example, a more-streamlined body shape may favor fast-swimming whales in escaping predation pressure, resulting in a slow life history strategy, while less-streamlined, slower swimming whales may specialize in foraging efficiency at the expense of predation mortality, leading to a fast life history strategy. Predation plays a significant role in shaping the life histories of prey species (Law, 1979; Walsh & Reznick, 2008), including odontocetes. Direct predation effects, such as changes in age/size-specific mortality, can have strong evolutionary consequences and influence the life history strategies of prey species (Benard, 2004; Jørgensen & Holt, 2013). For example, predation can impact growth rates and reduce the ability to procure food, indirectly affecting prey life histories (Lima, 1998; Matthews et al., 2020).

In many animal species, locomotion and foraging competition are critical for survival and reproductive fitness. However, there are trade-offs between traits that make an individual good at fleeing predators and traits that enhance foraging performance (Ludwig & Rowe, 1990). For example, rapid and economical swimming typically depends on a long, streamlined body shape and specialized muscles for the storage and recovery of elastic strain energy. This specialization in swimming performance may limit foraging efficiency. These biomechanical trade-offs likely contribute to the dichotomy between fast swimming and specialized foraging strategies observed in different phylogenetic lineages of cetaceans (Ford & Reeves, 2008). Some cetacean species may exhibit high performance in speed, allowing them to escape predation pressure through rapid swimming, while others may specialize in foraging efficiency at the expense of predation mortality, resulting in a slower life history strategy.

Foraging adaptations can influence life histories depending on the environmental conditions that either favor a sustained focus on energy acquisition to match a fast life or low energy acquisition foraging behavior that matches a slow life (Boggs, 1992; Pianka, 1976; Webb et al., 2003). In highly productive environments where food resources are abundant and nutrient-dense, animals may exhibit a fast life history strategy. They may focus on maximizing energy acquisition through behaviors such as specialized foraging techniques, efficient hunting strategies, or exploiting high-quality food sources. This can result in early maturation, short gestation periods, short interbirth intervals, and shorter lifespans, as the increased availability of food allows for a higher reproductive output and faster pace of life history events. Conversely, in food-insecure environments, where food resources are scarce or unpredictable, animals may adopt a slow life history strategy. They may have to invest more effort in acquiring food, such as through deep dives to forage for food, cooperative foraging behaviors, or seeking refuge habitats to avoid predators. These behaviors can lead to delayed maturation, longer gestation periods, longer interbirth intervals, and longer lifespans, as individuals may need to invest more in self-maintenance and survival before allocating energy to reproduction (Rogers & Smith, 1993). Understanding the interplay between foraging adaptations, predation conditions, and life history traits can provide valuable insights into the ecological and evolutionary dynamics of animals, including odontocetes.

Toothed whales (Odontoceti) are a particularly speciose group that include the main lineage families Delphinidae (dolphins), Monodontidae (beluga whale (*Delphinapterus leucas*) and narwhal (*Monodon monoceros*)), Phocoenidae (porpoises), Kogiidae (pygmy (*Kogia breviceps*) and dwarf (*K. sima*) sperm whales), Physeteridae (sperm whale, (*Physeter macrocephalus*)), and Ziphiidae (beaked whales) (McGowen, 2011). Odontocetes are characterized by a substantial range of body morphology and life histories, and thus represent a model group to test the hypothesis that predation/foraging morphology explains the evolution of fast/slow dichotomy. Within the Odontoceti suborder, different groups exhibit different phylogenetic origins, as well as unique morphological and ecological specializations. For example, crown-delphinids are approximately 10 My younger than crown-ziphiids (beaked whales). Delphinids exemplify a more-streamlined body shape and remarkable swimming speeds of up to 37 km/h, while ziphiids display a less-streamlined shape that may be related to their exceptional diving ability (Cozzi et al., 2010). The feeding behaviour of killer whales is known to vary across populations, with different groups targeting different prey and displaying unique behavioral patterns (Pitman & Ensor, 2003; Tavares et al., 2017). The primary predator of odontocetes is the killer whale, whose common name refers to their ability to kill and eat other whales (Heyning & Dahlheim, 1988). Fossil evidence of prey remains, skull morphology, tooth wear, and body size provide evidence that the ancestors of both killer and false killer whales (*Pseudorca crassidens*) had a fish-based diet up to around 1.3 Mya ago when these two distinct dolphin lineages independently evolved whale-eating diets (Berta et al., 2022; Bianucci et al., 2022; Ortega-Ortiz et al., 2014). Furthermore, predation pressure likely influenced the evolution of distinct life histories among different prey species (Cortés, 2000; Ferguson & Higdon, 2006; Forbes, 1993; Pagán et al., 2008).

Here we propose and test the hypothesis that evolutionary selection pressures are responsible for the divergent body shapes observed in odontocetes, and that these differences in morphology are directly or indirectly linked to specific life history strategies, which may explain the fast-slow dichotomy observed among cetaceans. Odontocetes may have evolved distinct body shapes to cope with predation pressure. Narrow fusiform body shapes may have evolved to maximize speed, allowing for escape from predators such as killer whales. In contrast, other species may have evolved more rotund body shapes that prioritize foraging ability, despite the associated increase in predation risk. This suggests that streamlined, fast whales are capable of outracing predators and have evolved slow reproductive life history, including late maturation, long gestation, long interbirth interval, and long life. Conversely, less-streamlined whales that are less able to escape killer whale predation through speed have had to rely on behavioural responses such as hiding at depths (Aguilar de Soto et al., 2020; Baird et al., 2008). As a result, these species have evolved life-history traits that ameliorate the demographic effects of predation. We hypothesize that less-streamlined whales invest more in their offspring, as evidenced by relatively large neonates, and exhibit accelerated timing of life-history events, including early maturation, short gestation, short interbirth interval, and short lifespan. We propose that the evolution of more streamlined body shapes in odontocetes occurred during the Pleistocene geological epoch which lasted from about 2,580,000 to 11,700 years ago, coinciding with the period when killer whales were evolving the behaviour, physiology, and morphology to hunt other whales as a food source (Bianucci et al., 2022). To test this hypothesis, we quantified the degree of streamlining in various odontocete species and examined the relationships between body shape and life history traits.

## Methods

First, we determined whether a whale species is more or less streamlined by performing a log-linear regression of body mass versus body length and categorized whales with positive residuals as ‘less-streamlined’, and those with negative residuals as ‘more-streamlined’. Next, we conducted ancestral reconstruction to compare the evolutionary history of more or less streamlined whales relative to the timing of the evolved ability of killer whales to prey on other whales. We then tested whether odontocete shape (with sample unit being whales) aligned with the previous clustering of cetaceans (including mysticetes) into reproducers and bet-hedgers (as established by Ferguson & Higdon, 2013), as well as the current clustering based solely on odontocetes. Additionally, we employed cluster analysis to group life-history traits (with the sample unit being traits) and utilized Principal Component Analysis (PCA) to generate factors representing these two groups, such as neonate length and temporal life-history traits. Subsequently, we employed linear mixed-effects models that accounted for phylogeny to investigate whether a causal relationship between whale shape and odontocete life-history traits could explain the observed variation.

### Data Management

Our nomenclature adheres to the guidelines set forth by the Society of Marine Mammalogy’s Committee on Taxonomy (2021). To update the odontocete life-history data, we compiled information from various sources, with a focus on published databases, while excluding river dolphins due to their limited exposure to killer whale predation and not being deep divers (Hamilton et al., 2001). The AnAge Database of Animal Ageing and Longevity, a component of the Human Ageing Genomic Resources (HAGR) project (Tacutu et al., 2018), served as the primary source for data on odontocete longevity, female age at sexual maturity, gestation length, interbirth interval, and neonate body length. To augment this, we also utilized data from the PanTHERIA database (Jones et al., 2009) for the same variables. For adult body mass, the EltonTraits database (Wilman et al., 2014) for cetacean species was employed as the primary source due to its larger sample size. Notable, all three databases are highly correlated and share the same values for many species for some variables, as the databases used similar sources. In total, we obtained data on species’ traits from 6 families, 24 genera, and 42 species of odontocete whales.

### Life-History Data

We selected neonate length (cm), age of maturity (y), gestation length (d), interbirth interval (y), and longevity (y) as the key life-history variables. To ensure data quality, we only included species with length and mass data for at least 5 adult individuals. We did not include nursing duration due to uneven data quality leaving us with complete data for 19 of the 42 species. For the 23 whales with missing values (13% of trait values missing), we substituted a residual value of zero from a linear regression of body length on each life-history trait, following the approach of Weijerman et al., (2005). This minimized the impact of missing values on the statistical tests and allowed us to maximize statistical power by including all odontocete species. To normalize the distribution of data, which exhibited a strong right skew, all variables were log-transformed, a standard practice in comparative approaches (Ives & Garland, 2014). Normality of all log10-transformed data distributions was confirmed using Wilk-Shapiro normality tests for all traits.

To assess body shape, we calculated an index based on residuals from a log–log regression of body mass versus body length. Positive residuals were assigned to whales deemed less streamlined, while negative residuals were assigned to whales considered more streamlined. Residual values closer to zero indicated greater uncertainty in the designation of streamlined body types, whereas larger magnitude differences indicated a clearer categorization into either more or less streamlined body shapes. This approach allowed for a continuum of body shapes, with the magnitude of the residuals providing an indication of the degree of streamlining in each whale species.

### Ancestral Reconstruction

**Fig. 1:**
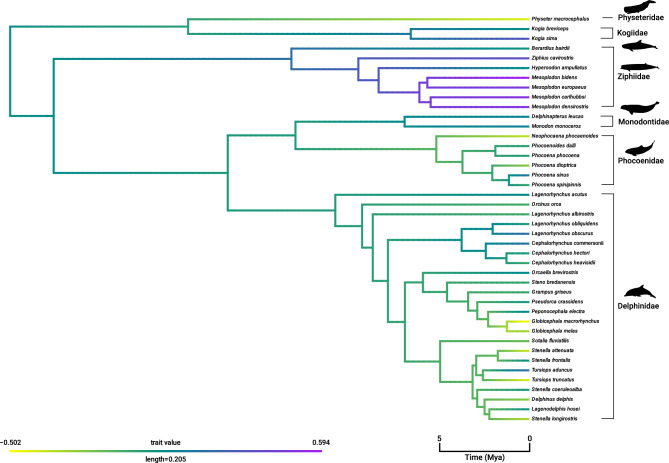
Phylogenetic reconstruction of body shape evolutionary history of odontocete family. Negative values indicate more-streamlined whales and positive trait values indicate less-streamlined body shape

Ancestral reconstruction is a statistical method that uses phylogenetic inference to construct an evolutionary tree, or “phylogeny”, representing the evolutionary relationships between species (Joy et al., 2016). Here, we used phylogenetic inference to construct a phylogeny that represented the evolutionary relationships among odontocete species. We developed a phylogeny for the 42 odontocete species based on McGowen et al., (2020) to control for phylogenetic effects and to understand the evolutionary history of more- or less-streamlined whales (Fig. 1). Ancestral reconstruction was performed using the Ancestral Character Estimation (ace) method in the ape package (version 5.5), which estimated the characteristics of ancestral species based on the characteristics of their descendants, while also accounting for uncertainty (Garamszegi & L.Z. ed., 2014). Maximum likelihood values at a given node were computed using only the information from the tips and branches descending from that node, projecting the phylogenetic tree in a space defined by phenotype (on the y-axis) and time (on the x-axis) (Evans et al., 2009).

### Grouping Whales and Life-History Traits

We conducted a hierarchical cluster analysis to test whether more or less streamlined odontocetes exhibited differences in life-history traits similar to the previously reported dichotomy (Ferguson & Higdon, 2013) but here, we excluded mysticetes as a group due to their different life history (Wade et al., 2012). We used a binomial generalized linear model (GLM) with a logistic link to test for differences in whale shape, which was categorized as 0 for less streamlined and 1 for more streamlined. We also performed a cluster analysis of life-history traits using a Pearson's chi-square test with Yates' continuity correction to assess the similarity between the cluster analysis results.

Species-mode cluster analysis examined clusters of objects (species) for a measured number of variables (life-history traits), and the variable values were used to measure distance. Cluster analysis assigned species as similar according to the average Euclidean distance between clusters using hclust (version 2.1.3; Maechler et al., 2022). Analyses used the unpaired group averaging method, which had the highest cophenetic correlation coefficient compared with other cluster analysis methods (Romesburg, 1984).

To model statistical relationships between whale shape (measured by body length and mass) and temporal life-history traits (n = 4), we needed to reduce the number of explanatory variables due to our relatively small sample of 42 species (Knofczynski & Mundfrom, 2008). In addition, the life-history traits were strongly collinear (Supplementary Fig. 1). Therefore, we conducted a trait-mode cluster analysis of life history data to reduce the five traits to a smaller number of non-correlated explanatory variables (Romesburg, 1984). The cluster analysis examined clusters of variables (temporal life-history traits), with object values (species) measuring the distance between variables. This reduction in the number of explanatory variables helped to interpret variable contributions relative to multivariate analyses (see below).

### Relationships Among Variables

We assessed whether phylogenetic signal was present in odontocete life-history traits, which refers to the pattern where closely related species exhibit more similar trait values than more distantly related species. We tested whether phylogenetic corrections were necessary to account for the potential violation of the statistical assumption of independence due to phylogenetic structure, following the methods of Harvey and Pagel (1991) and Freckleton (2000). We estimated phylogenetic signal using Pagel's λ (Pagel, 1994) and Blomberg's K (Blomberg et al., 2003) methods, implemented using the treeplyr (Harmon, 2020) and phytools (Revell, 2012) packages in R.

To control for body-size effects, we calculated residuals for the life-history traits. We then used mixed-effects models with a Brownian correlation structure to control for phylogeny and determine the relationship between whale shape and life-history traits, following the methods of Harvey and Keymer (1991) and Martins and Hansen (1997). Finally, to compare models with nested fixed effects while controlling for phylogeny, we used maximum likelihood (ML) estimation, as described by Zuur et al. (2009).

Taxonomic identifiers for odontocete species names (scientific & common) and taxonomic hierarchical information were obtained from the National Center for Biotechnology Information (NCBI) Taxonomy database (Federhen, 2012) and we adopted the phylogeny and branch lengths for the analyses from McGowen et al. (2020). Assembled taxonomic data were used to control for phylogeny and perform phylogenetic signal testing in the analyses of the life-history traits and their relationship with whale shape (Chamberlain & Szöcs, 2013).

## Results

### Data Management

**Fig. 2:**
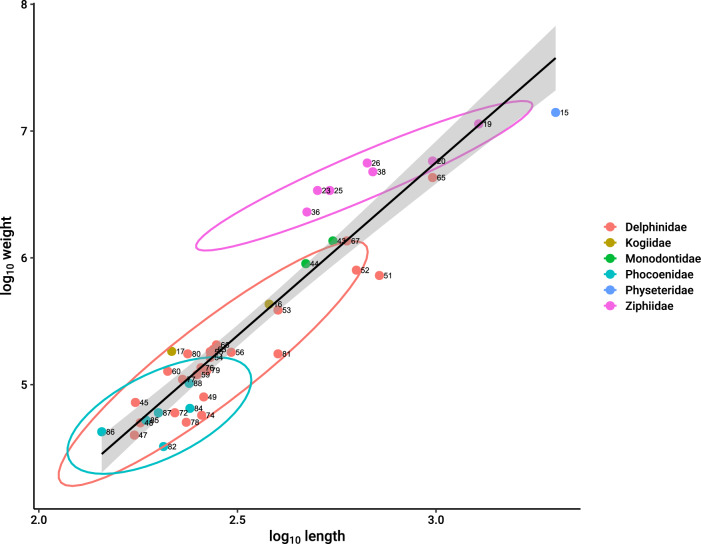
Log–log regression of odontocete mass versus length describing body shape of whales belonging to different families. Note that Ziphiidae whales are less-streamlined in shape and Phocoenidae are more streamlined in shape

Odontocete whale body mass spanned three orders of magnitude from Indo-Pacific finless porpoise (*Neophocaena phocaenoides*) at 32.5 kg–14,025 kg sperm whale (*Physeter macrocephalus*) (Table 1). The residuals of log mass versus log length, obtained from linear regression, were used to assess body shape, with positive values indicating less streamlined and negative values indicating more streamlined whales (Table 2). Beaked whales were generally found to be less streamlined, while porpoises (Phocoenidae) were more streamlined, and dolphins (Delphinidae) fell within the average range for odontocetes (Fig. 2).

**Table 1:** Life-history information for odontocete whale species

Family	Species	Common name	Group	Length	Mass	Gest	Inter	Long	Neolen	ASM
Physeteridae	*Physeter macrocephalus*	Sperm whale	B	2000	14,025,000	15	60	77	387.5	9
Kogiidae	*Kogia breviceps*	Pygmy sperm whale	R	380	431,500	10	12	17	124.8	**8.03**
Kogiidae	*Kogia sima*	Dwarf sperm whale	NA	216	183,067	9.1	**25.5**	23	100	5
Ziphiidae	*Berardius bairdii*	Baird’s beaked whale	B	1280	11,380,000	17	24	84	460	12
Ziphiidae	*Hyperoodon ampullatus*	Northern bottlenose whale	R	980	5,800,000	12	24	37	300	10
Ziphiidae	*Mesoplodon carlhubbsi*	Hubbs’ beaked whale	NA	540	3,400,000	12	**34.8**	**46.4**	249	**8.92**
Ziphiidae	*Mesoplodon europaeus*	Gervais’ beaked whale	NA	671	5,600,000	**13.5**	**37.5**	48	210	**9.52**
Ziphiidae	*Mesoplodon densirostris*	Blainville’s beaked whale	R	473	2,300,000	**12.8**	**33.3**	27	190	9
Ziphiidae	*Ziphius cavirostris*	Cuvier’s beaked whale	R	693	4,775,000	12	**37.9**	62	270	9
Monodontidae	*Delphinapterus leucas*	Beluga whale	B	550	1,360,000	14.5	36	77	150	9
Monodontidae	*Monodon monoceros*	Narwhal	B	470	900,000	15.3	36	115	160	6.5
Delphinidae	*Cephalorhynchus commersonii*	Commerson’s dolphin	R	175	72,400	11.5	**23.8**	18	65	7.5
Delphinidae	*Cephalorhynchus heavisidii*	Heaviside’s dolphin	R	174	40,000	10.5	**23.8**	**21.2**	85	7.5
Delphinidae	*Cephalorhynchus hecto*ri	Hector’s dolphin	R	180	50,000	10.5	36	20	60	7.5
Delphinidae	*Delphinus delphis*	Common dolphin	R	260	80,000	10.5	24	22	85	7.5
Delphinidae	*Globicephala macrorhynchus*	Short-finned pilot whale	B	720	726,000	15	84	63	185	9
Delphinidae	*Globicephala melas*	Long-finned pilot whale	B	630	800,000	12	42	60	176	12
Delphinidae	*Grampus griseus*	Risso’s dolphin, grampus	R	400	387,500	12	**31.4**	42.5	135	**8.16**
Delphinidae	*Lagenodelphis hosei*	Fraser’s dolphin	R	270	164,000	12	24	17.5	100	7
Delphinidae	*Lagenorhynchus acutus*	Atlantic white-sided dolphin	NA	270	182,000	10.5	12	27	120	9
Delphinidae	*Lagenorhynchus albirostris*	White-beaked dolphin	R	305	180,000	12	**28.7**	**31.2**	115	8
Delphinidae	*Lagenorhynchus obliquidens*	Pacific white-sided dolphin	R	250	120,000	12	**26.8**	46	102.5	7.5
Delphinidae	*Lagenorhynchus obscurus*	Dusky dolphin	R	211	127,500	13	28.6	**24.2**	70	4.5
Delphinidae	*Orcaella brevirostris*	Irrawaddy dolphin	R	275	190,000	14	**27.7**	30	100	5
Delphinidae	*Orcinus orca*	Killer whale, orca	B	980	4,300,000	16.5	60	90	240	15
Delphinidae	*Peponocephala electra*	Melon-headed whale	B	280	206,000	12	**27.9**	47	100	12
Delphinidae	*Pseudorca crassidens*	False killer whale	B	596	1,360,000	15.5	84	62.5	160	11
Delphinidae	*Sotalia fluviatilis*	Tucuxi	R	220	60,000	11.5	22.5	35	95	**6.82**
Delphinidae	*Stenella attenuata*	Pantropical spotted dolphin	B	257	57,400	11.5	30	46	83	10
Delphinidae	*Stenella coeruleoalba*	Striped dolphin	B	256	135,900	12.5	40	57.5	96.5	9
Delphinidae	*Stenella frontalis*	Atlantic spotted dolphin	R	230	110,000	12	36	**25.7**	104	11.5
Delphinidae	*Stenella longirostris*	Spinner dolphin	NA	235	50,500	10.5	36	**26.1**	80	5.5
Delphinidae	*Tursiops aduncus*	Indo-Pacific bottlenose dolphin	R	237	175,000	12	36.5	**26.2**	88	9
Delphinidae	*Tursiops truncatus*	Common bottlenose dolphin	B	400	175,000	12	24	51.6	117	9
Phocoenidae	*Neophocaena phocaenoides*	Indo-Pacific finless porpoise	R	206	32,500	11	24	34	77	7
Phocoenidae	*Phocoena dioptrica*	Spectacled porpoise	B	240	65,000	**11.4**	**26.5**	8	94	3
Phocoenidae	*Phocoena phocoena*	Harbor porpoise	R	186	52,500	10.5	12	20.4	70	3.5
Phocoenidae	*Phocoena sinus*	Vaquita	R	144	42,500	10.5	**22.3**	21	74	**6.01**
Phocoenidae	*Phocoena spinipinnis*	Burmeister’s porpoise	B	200	60,000	11.5	**24.9**	8	86	**6.63**
Phocoenidae	*Phocoenoides dalli*	Dall’s porpoise	NA	239	102,500	11.4	30	22	100	5
Ziphiidae	*Mesoplodon bidens*	Sowerby’s beaked whale	R	503	3,400,000	12	**34**	**44.1**	**102.5**	**8.73**
Delphinidae	*Steno bredanensis*	Rough-toothed dolphin	B	265	130,000	**11.1**	**27.4**	32	**70**	10

Data in bold were interpolated from log–log residual regression analysis (see Methods). *Group* from Ferguson and Higdon (2010), *Length* Adult body length (cm), *Mass* Adult body mass (g), *Gest* Gestation length (mo), *Long* Longevity (y), *Neolen* Neonate body length (cm), *ASM* Age of sexual maturity (y)

**Table 2:** Log linear regression results of odontocete whale body length versus life-history traits

Dependent variable (log10)^a^	Intercept	Coefficient (slope)	Adj R^2^	df	F-statistic	P
Body mass	− 1.446 ± 0.400***	2.733 ± 0.157***	0.884	1,40	304.3	< 0.001
Neonate length	0.1219 ± 0.0986	0.775 ± 0.0386***	0.914	1,38	403.1	< 0.001
ASM^b^	0.1338 ± 0.2214	0.2999 ± 0.0866***	0.271	1,32	11.92	0.002
Gestation length	0.162 ± 0.027***	0.6712 ± 0.0699***	0.494	1,36	35.19	< 0.001
Interbirth interval	0.3378 ± 0.1439	0.6186 ± 0.3736	0.193	1,23	5.51	0.028
Longevity	− 0.2231 ± 0.3389	0.6922 ± 0.1315***	0.464	1,32	27.7	< 0.001

*** probability <0.001

^a^Independent variable log10(length)

^b^Age of sexual maturity

### Ancestral Reconstruction

The analysis of the odontocete traits revealed that six out of seven traits, including five temporal and two morphological traits, exhibited a significant phylogenetic signal. This indicates that consideration of phylogeny is necessary in any statistical analysis involving these traits. The morphology traits, specifically adult body length (λ = 0.99, K = 1.12), adult mass (λ = 0.99, K = 1.74), and neonate length (λ = 0.97, K = 1.28), showed the strongest phylogenetic signals. Gestation length (λ = 0.916, K = 0.374) and age of sexual maturity (λ = 0.652, K = 0.318) also exhibited significant phylogenetic signals, albeit comparatively weaker. The only trait that did not show a significant phylogenetic signal was interbirth interval (λ = 0.445, K = 0.232). Therefore, in statistical analyses involving all life-history traits, phylogenetic corrections were applied to account for the phylogenetic structure of the data.

### Grouping Whales and Life-History Traits

**Fig. 3:**
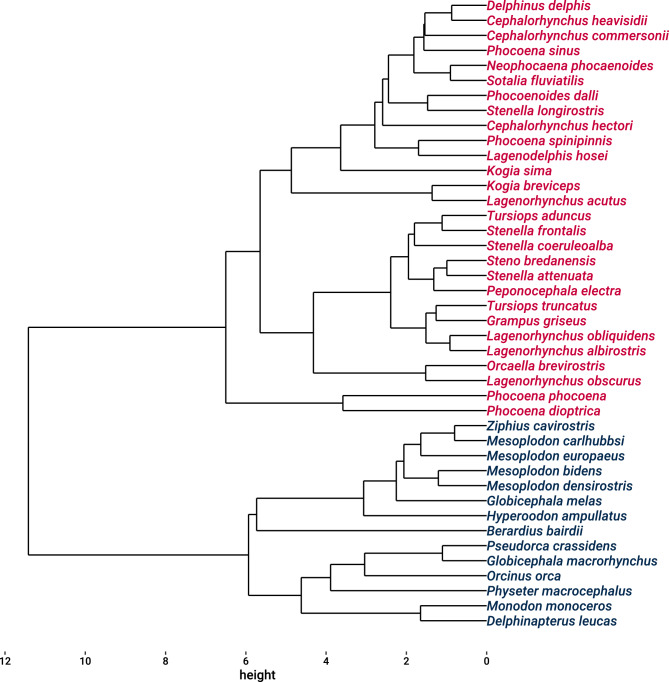
Cluster analysis of odontocete whales according to life-history traits resulting in two groupings – one group (red) characterized by slow life history and another (black) with fast life history (i.e., early age of sexual maturation, short gestation, short interbirth interval, and short life)

Species-mode cluster analysis resulted in two groups of whales. The first group consisted of 14 whales and included many of the less-streamlined whales, such as beaked whales. The second group consisted of 28 whales and included many of the dolphins and porpoises (Fig. 3). This resulted in two different groupings of odontocetes based on life-history traits: (1) the previous clustering of cetaceans (Ferguson & Higdon, 2013) excluding the Mysticetes, and (2) the current clustering based on a more comprehensive set of life-history traits for odontocetes. A comparison of the previous clustering with the current clustering found a different pattern (Chi-square = 1.44, df = 1, p = 0.23).

Logistic regression of the previously identified whale clusters (Ferguson & Higdon, 2013) with whale shape showed a significant pattern (z = 2.060, p = 0.032, 35 df), with less-streamlined whales being more likely to be reproducers and more-streamlined whales being more likely to be bet-hedgers. Specifically, with every one-unit increase in the shape index (residual value of log(mass) ~ log(length)), the odds of being a reproducer increased 53-fold. However, when comparing the more or less streamlined whales to the cluster analysis of the updated odontocete life-history traits, no significant relationship was found (z = 1.630, p = 0.1031, Null deviance = 53.467 with 41 df).

**Fig. 4:**
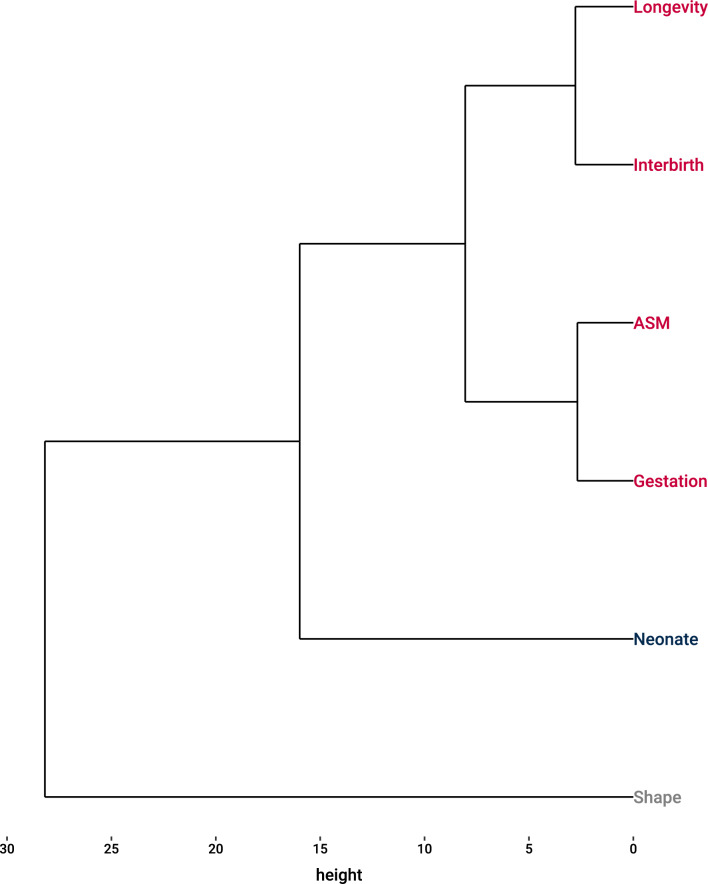
Cluster analysis of life-history variables for odontocete whales describing two groups: (1) associated with morphology (neonate body length) and (2) associated with temporal life-history traits (age of sexual maturity, gestation length, interbirth interval, longevity). Note that whale shape (residuals from log–log regression of whale mass versus length) clusters as an outlier indicating little association with life-history traits

Next, the trait-cluster analysis separated body length and body mass from temporal life-history traits, with body shape remaining as an outlier (Fig. 4). As a result, we ran a PCA analysis on the four temporal life-history traits (age of maturity, gestation length, interbirth interval, and longevity) to reduce the number of explanatory variables.

### Relationships Among Variables

**Fig. 5:**
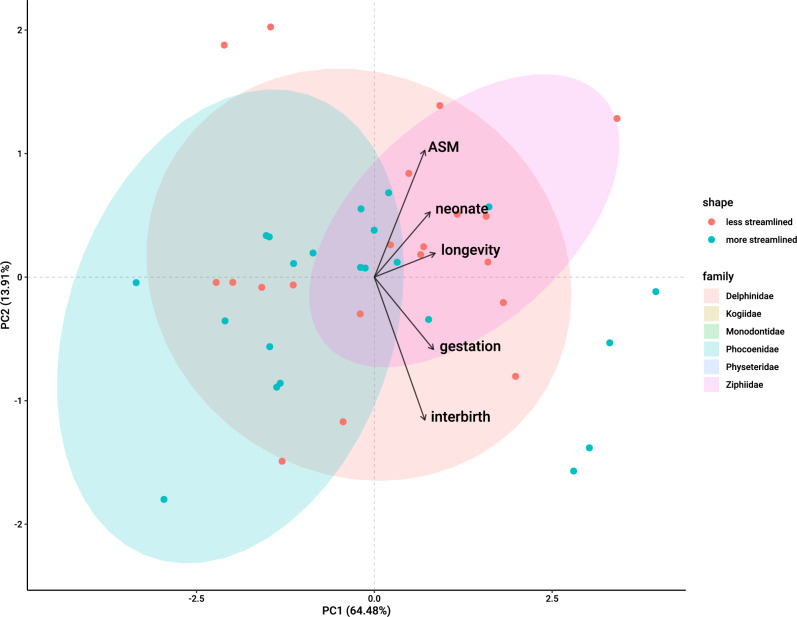
Principal component analysis results illustrating clustering of odontocete whale temporal life-history traits by family according to the two top vectors that explain 83% of the variation (PC1 64.48% and PC2 13.91%). Note that the Ziphiidae family (beaked whales) and Phocenidae (porpoises) are characterized by a dissimilar life histories along PC1. Ellipses represent 90% confidence interval for core family life-history traits. The vectors in blue represent the life-history traits that contributed above expected average (12.5%) to PCA

The PCA analysis reduced the variables to two vectors. The first vector (PC1) was positively correlated with all four temporal life-history traits, including age of sexual maturity, gestation length, interbirth interval, and longevity, with similar axis loadings for all traits (Table 3). On the other hand, PC2 was most strongly correlated with age of sexual maturity and negatively correlated with gestation length and interbirth interval. Graphical evidence from the PCA plot did not suggest a clear separation of whales based on body shape. However, visual examination of the plot suggested that size variables, such as adult and neonate length, were positively related to shape, while temporal life-history traits were negatively related to shape (Fig. 5).

**Table 3:** (A) Results of principal components analysis (PCA) of odontocete temporal life-history traits. The resulting two vectors explained 82% of the variance of the four life-history traits. (B) PCA results by odontocete family

(A)Log (variable)	PC1	PC2
Gestation length	0.524	− 0.357
Interbirth interval	0.482	− 0.511
Longevity	0.540	0.168
Age of Sexual maturity	0.449	0.764
Proportion of variance	0.666	0.167
Standard deviation	1.632	0.817

(B)Family	Shape (+ = less streamlined,—= more streamlined)	PC1	PC2
Delphinidae	− 0.0837	0.242	− 0.451
Kogiidae	+ 0.1793	1.569	1.953
Monodontidae	+ 0.0936	− 2.344	− 0.0248
Phocoenidae	− 0.0969	2.233	0.6334
Physeteridae	− 0.4289	− 5.107	0.964
Ziphiidae	+ 0.3536	− 2.597	1.473

Controlling for body size, mixed-effects models that tested for relationships between life-history traits and body shape, while accounting for phylogeny and body size, revealed a positive association between shape and neonate length, but no significant relationships with either principal component (Table 5). Additionally, the principal components for temporal life-history traits, including age of sexual maturity, gestation length, interbirth interval, and longevity, did not significantly correlate with body shape (Table 5). It should be noted that some of the life-history traits were constrained by seasonality, which limited the variation. For example, most gestational and interbirth intervals were close to annual (Table 1).

Furthermore, when controlling for body size, life-history traits varied among families, except for interbirth interval (Table 4). As observed in the cluster analysis, porpoises were generally more streamlined and presumed to be fast, while beaked whales were less streamlined. However, the mixed-effects models did not find significant relationships between life-history traits and body shape after accounting for phylogeny and body size (Table 5).

**Table 4:** Life-history variables used in analyses summarized by odontocete family (mean ± standard deviation (sample size))

Family	Measure	Shape	Length (cm)	Mass (kg)	Neonate (g)	ASM (y)	Gestation (mo)	Interbirth (mo)	Longevity (y)
Delphinidae	Unadjusted Adjusted	− 0.0838 ± 0.193	336 ± 199 (24)	422 ± 901 (23)	111.4 ± 42.9 (23) − .504 ± 0.0511	8.8 ± 2.5 (22) + 0.0575 ± 0.107	12.2 ± 1.6 (23) + 0.0156 ± 0.0468	38.7 ± 20.7 (16) 0.0631 ± 0.173	42.6 ± 19.5 (18) + 0.0575 ± 0.121
Kogiidae	Unadjusted Adjusted	+ 0.179 ± 0.211	298 ± 116 (2)	307 ± 175 (2)	112.4 ± 17.5 (2) + 0.0439 ± 0.0695	5.0 (1) − 0.0709 ± 0.1000	9.6 ± 0.6 (2) − 0.0213	12.0 (1) − 0.184 ± 0.260	20.0 ± 4.2 (2) − 0.176 ± 0.200
Monodontidae	Unadjusted Adjusted	+ 0.0936 ± 0.00514	510 ± 57 (2)	1130 ± 325 (2)	155.0 ± 7.1 (2) − 0.0670 ± 0.0581	7.8 ± 1.8 (2) − 0.0222 ± 0.0915	14.9 ± 0.6 (2) + 0.0949 ± 0.0186	36.0 ± 0.0 (2) + 0.101 ± 0.00319	96.0 ± 26.9 (2) + 0.347 ± 0.153
Phocoenidae	Unadjusted Adjusted	− 0.0969 ± 0.188	203 ± 36 (6)	59 ± 24 (6)	83.5 ± 11.9 (5) + 0.0977 ± 0.0423	4.6 ± 1.8 (4) − 0.133 ± 0.165	11.0 ± 0.5 (5) − 0.0161 ± 0.0166	22.0 ± 9.2 (3) − 0.0587 ± 0.144	18.9 ± 9.8 (6) − 0.156 ± 0.276
Physeteridae	Unadjusted Adjusted	− 0.429 (1)	2000 (1)	14,025 (1)	387.5 (1) − 0.107	9.0 (1) − 0.0554	15.0 (1) + 0.0718	60.0 (1) + 0.284	77.0 (1) − 0.108
Ziphiidae	Unadjusted Adjusted	+ 0.354 ± 0.237	734 ± 295 (7)	5236 ± 2993 (7)	279.8 ± 96.8 (6) + 0.485 ± 0.0600	10.0 ± 1.4 (4) + 0.0324 ± 0.0384	13.0 ± 2.2 (5) + 0.0171 ± 0.0520	24.0 ± 0.0 (2) − 0.0279 ± 0.047	51.6 ± 22.3 (5) − 0.0424 ± 0.118
ANOVA test	Adjusted	F_5,36_ = 6.94, p < 0.001			F_5,36_ = 6.94, p < 0.001	F_5,36_ = 3.38, p = 0.013	F_5,36_ = 4.44, p = 0.003	F_5,36_ = 2.16, p = 0.081	F_5,36_ = 4.60, p = 0.002

Adjusted values control for body size using residuals

**Table 5:** Linear effects model results of dependent variable shape (residuals of log(mass) ~ log(length)) using standard regression and controlling for phylogenetic effects

		Standard	Regression^1^		Phylogeny	Controlled	Regression^2^	
Coefficient	Estimate	Standard Error	t value	p-value	Estimate	Standard Error	t value	p-value
Intercept	0.00284	0.0351	0.0809	0.936	0.0420	0.105	0.401	0.691
RNL†	2.338	0.601	3.891	0.0004	1.419	0.596	2.382	0.0224
PC1‡	0.0109	0.0223	0.490	0.627	− 0.0218	0.0403	− 0.540	0.593
PC2‡	− 0.0115	0.0448	− 0.256	0.799	− 0.0119	0.0238	− 0.500	0.620

Explanatory variables include neonate body length and Principal Component Analysis (PCA) vectors that combined four temporal life-history traits

^†^Residuals neonate length

^‡^PCA variable from four temporal life history traits – age of sexual maturity, gestation length, interbirth interval, and longevity

^1^Generalized least squares fit by REML (shape ~ neonate length + PC1 + PC2); AIC = 15.11; BIC = 23.30; Log likelihood = -2.56; Residual standard error = 0.228; df 42 total (38 residual)

^2^Controlling for phylogeny generalized least squares fit by REML: AIC = 10.13; BIC = 19.96; Log likelihood = 0.934; Correlation Structure using Pagel statistic (lambda = 0.582);

Residual standard error: 0.263; Degrees of freedom 42 total (38 residual)

## Discussion

Our study is the first to use ancestral trait reconstruction to investigate the evolution of body shape and its relationship to life history traits in odontocetes. Life-history theory recognizes the duality of the fast-slow continuum (Bielby et al., 2007), and evolutionary ecology acknowledges that food competition and predation are two environmental selection pressures affecting reproduction and survival (Murphy, 1968; Walsh & Reznick, 2009; Wilbur et al., 1974). In this study, we investigated whether whale morphology, specifically the degree of streamlining, is aligned with the fast-slow life-history continuum. It has been suggested that odontocete whales with more fusiform body shapes, enabling greater speed, can minimize killer whale predation (Domenici, 2001; Ford et al., 2005). Therefore, we hypothesized that these more-streamlined whales would have evolved (1) reduced investment in offspring, indicated by smaller neonates, and (2) a slower timing of life-history events, such as delayed sexual maturation and longer gestation length, interbirth interval, and longevity. Conversely, we predicted that less-streamlined species that evolved enhanced foraging features at the expense of speed would (1) invest more energy in progeny, leading to larger neonate body size, and (2) reduce the timing of life-history events, as indicated by earlier age of sexual maturity and shorter gestation length, interbirth interval, and longevity. Our results showed the predicted relationship between body shape and neonate size, but we did not find a relationship between body shape adaptations and fast or slow life histories. Although morphological adaptations associated with speed may have evolved among odontocetes in response to killer whale predation, life-history traits related to the timing of life-history events have not.

Ancestral reconstruction suggests that many odontocetes evolved more streamlined body shapes in response to the evolution of killer whale that allowed them to catch and eat other whales approximately 1 Mya (Kurtén, 2017). Although oceanic predators of cetaceans existed since the divergence of Odontocetes from Mysticetes around 30 Mya (Thewissen & Williams, 2002), many of the larger predators, such as *Otodus megalodon *(Shimada et al., 2016; Pimiento et al., 2017; Cooper et al., 2022) and a large physeteroid (sperm whale) (Kimura et al., 2006; Lambert et al., 2014; Peri et al., 2022) disappeared around the end of the Pliocene, a period marked by climatic variability and sea-level fluctuations (Pimiento et al., 2017). The earliest known fossil of a killer whale, *O. citonensis*, dates back to the Pliocene Epoch (5.3 million to 2.6 Mya) and was only about 4 m in length, similar in size to a typical dolphin (Galatius et al., 2020). This suggests that during the Pleistocene, prior to the evolution of larger-bodied killer whales that evolved to eat other odontocetes (Berta et al., 2022), there may have been a lack of large oceanic predators of odontocetes. The split between the ancestors of modern killer whales and their closest living relatives, the false killer whales, occurred around 1.9 million years ago with a large margin of error (range of 700,000 to 3.5 Mya) (Foote et al., 2013).

Fusiform body shape in odontocetes appears to have evolved recently, with extremes in body shape associated with speed emerging during the Pleistocene (< 2.5 Mya), a period characterized by repeated glaciations (Fig. 1). This pattern is particularly evident in dolphins (family Delphinidae) and porpoises (family Phocoenidae), which tend to be more streamlined, while beaked whales have retained a less-streamlined body shape (Fig. 1). However, there does not appear to be a corresponding shift in life-history traits among odontocetes.

Beaked whales are known for their less-streamlined bodies, characterized by a small dorsal fin and short narrow flippers. Despite their relatively slow swimming speeds, most beaked whale species have relatively fast life histories when their larger size is taken into account (Table 4). These whales are deep divers, often feeding entirely on squid (MacLeod, 2018) and foraging at extreme depths may have placed constraints on their morphology (Peters et al., 2022), potentially explaining their less-streamlined body shape. Foraging styles among beaked whales typically involve slow, energy-conserving movements during long, deep dives, with reproduction requiring energy-dense prey and high-quality habitat to support survival and reproduction (New et al., 2013). These factors likely contributed to the evolution of beaked whale life-history strategies (Feyrer et al., 2020).

Pelagic species belonging to the Delphinidae family are characterized by their streamlined body shape, allowing them to swim at high speeds (Curren et al., 1994). Despite the faster swimming ability of killer whales and false killer whales (*Pseudorca crassidens*) compared to common bottlenose dolphins (*Tursiops truncatus*), the smaller dolphins display greater mobility and are able to swim at higher relative speeds, which enhances their ability to escape predation (Fish, 1998). In addition to body shape, the fluke design of dolphins also contributes to their superior swimming performance. Although the precise dates of morphological evolution remain uncertain, many dolphins appear to have evolved their streamlined body shape during the Pleistocene geological epoch (< 2.5 Mya), which coincides with the time killer whales developed their hunting ability to prey on other whales (Pyenson, 2017). This shift led to the evolution of a more streamlined morphology in many odontocete whales, likely as an adaptation to contend with the new predator. Dolphins have a slow life history indicated by their long lifespan and relatively slow prenatal growth relative to their body size (Huang et al., 2008). Furthermore, they are considered income breeders, relying on energy acquired during the reproductive period (Huang et al., 2011).

Our analysis revealed that morphological and temporal life-history traits clustered, which supports the hypothesis of correlated traits functioning as genetic modules (Murren, 2012). Correlated selection has been proposed in previous studies (Kelly, 1992; Santos et al., 2021), and research on mammals has demonstrated that temporal life-history traits can either be extended or shortened in response to selection for reproductive adaptations such as delayed implantation (Ferguson et al., 1996), diet (Fisher et al., 2001), or maximum lifespan (Mayne et al., 2019). Understanding the underlying mechanisms that cause these traits to cluster and show coordinated evolutionary changes may be useful in developing species response models to global climate change (Waldvogel et al., 2020). By incorporating an evolutionary perspective on the limits of adaptive genetic change for a species, we can better predict their ecological flexibility in response to changing conditions. For instance, a slow species may lack the genetic capacity to exploit the benefits of greater primary productivity resulting from warming (Cheung et al., 2008).

We limited our analysis to odontocetes, excluding other marine mammal groups like mysticetes as the former exhibit a range of responses to killer whale predation, including hiding and maneuverability (Domenici, 2002; Matthews et al., 2020). They also display variability in life history strategies (Busson et al., 2019; Ferguson et al., 2012; Morisaka & Connor, 2007). Mysticetes have fewer species than Odontocetes and therefore are not as useful for phylogenetic studies, but as a group they may represent multiple approaches to defending against killer whale attacks, which could lead to life-history evolutionary responses to predation pressure (Corsi et al., 2022; Ford & Reeves, 2008).

While our study focused on odontocete shape and its correlation with swimming speed (Fish, 1998), the relationship between shape and life history has not been explored. We recognize that other factors besides speed, such as habitat, evolutionary history, and social considerations, can also influence the prey’s ability to evade predators (Scherer & Smee, 2016). Group size may be a factor in social species (Blumstein, 2006), but the influence of sociality on life-history evolution may decouple from the evolutionary response of shape. There is taxonomic uncertainty among odontocete families, particularly within delphinids, and new beaked whale species have been identified (Dalebout et al., 2002; LeDuc et al., 1999). The ancestral state reconstruction produced in our study does not fully consider the diversity of cetacean body shapes or predators over mammalian history. We recommend additional research on taxonomy and the evolution of odontocetes. Other phylogenetic groups should be studied to test whether morphology matches the life-history pattern. Our study focused on the impact of *Orcinus* predation pressure on the evolution of body proportions in odontocetes; however, other factors such as adaptation to long-distance migration or sexual selection may also have influenced the evolution of body shape in some odontocete clades. We have not evaluated these competing hypotheses.

A significant consideration for conservation is the need for research that includes knowledge of evolutionary trends in ecology (e.g., morphology and predation) and life history (e.g., longevity) to mitigate the threats of climate change. Odontocetes provide a useful taxonomic group for assessing evolution and conservation since they exhibit a wide range of morphology and life history, and play both predator and prey roles (Rupil et al., 2022). Although we did not find a strong link between shape and life history in this study, further research could investigate other explanations for the fast-slow life-history continuum in odontocetes. For example, environmental selection pressure may explain the dichotomy with offshore species evolving slower temporal life-history traits relative to inshore species (Crawford et al., 2006; Rolland et al., 1998; Whitfield, 1990). In addition to studying predation effects, investigations are required into how foraging mode influences life history traits such as growth, maturity, reproduction, and survival. Future research should examine the morphological evolution of the fast-swimming odontocetes beyond a simple length-mass relationship. This research could include investigating differences in evolved appendage morphology, such as fore flippers and flukes that may increase agility at the expense of speed, thereby allowing for defense against killer whale predation (Adamczak et al., 2020; Scheffer, 1952). It is possible that the intense selection pressure of recently evolved killer whale predation favored fast-swimming morphological adaptations in their prey during a predator–prey race. However, it is also possible that behavioral and life-history traits may take longer to evolve in step with morphology. An understanding of life-history evolution will assist in identifying species that are less able to adapt to the effects of climate change and, therefore, require more significant conservation efforts (Nicotra et al., 2015).

## Supplementary Information

Below is the link to the electronic supplementary material.

Supplementary file1 (DOCX 433 KB)

## Data Availability

Raw data used in this study are provided in Table 1, and results from data analyses are available from the corresponding author upon reasonable request.
