# Supplementary material for: Investigating the Relationship Between Body Shape and Life History Traits in Toothed Whales: Can Body Shape Predict Fast-Slow Life Histories?
Source: Evol Biol. 2023 May 23;50(3):300–17. doi: 10.1007/s11692-023-09605-4 (PMC10415445; doi:10.1007/s11692-023-09605-4)
Supplement: Supplementary file 1 — Supplementary file1 (DOCX 433 KB) [file 11692_2023_9605_MOESM1_ESM.docx]

Supplementary Figure 1. Correlation (R^2^ from -1.0 to +1.0) of morphological (body length, mass, body shape from regression) and temporal life-history traits (longevity, neonate length, interbirth interval, gestation length, age of sexual maturity) for 42 Odontocete whale species.


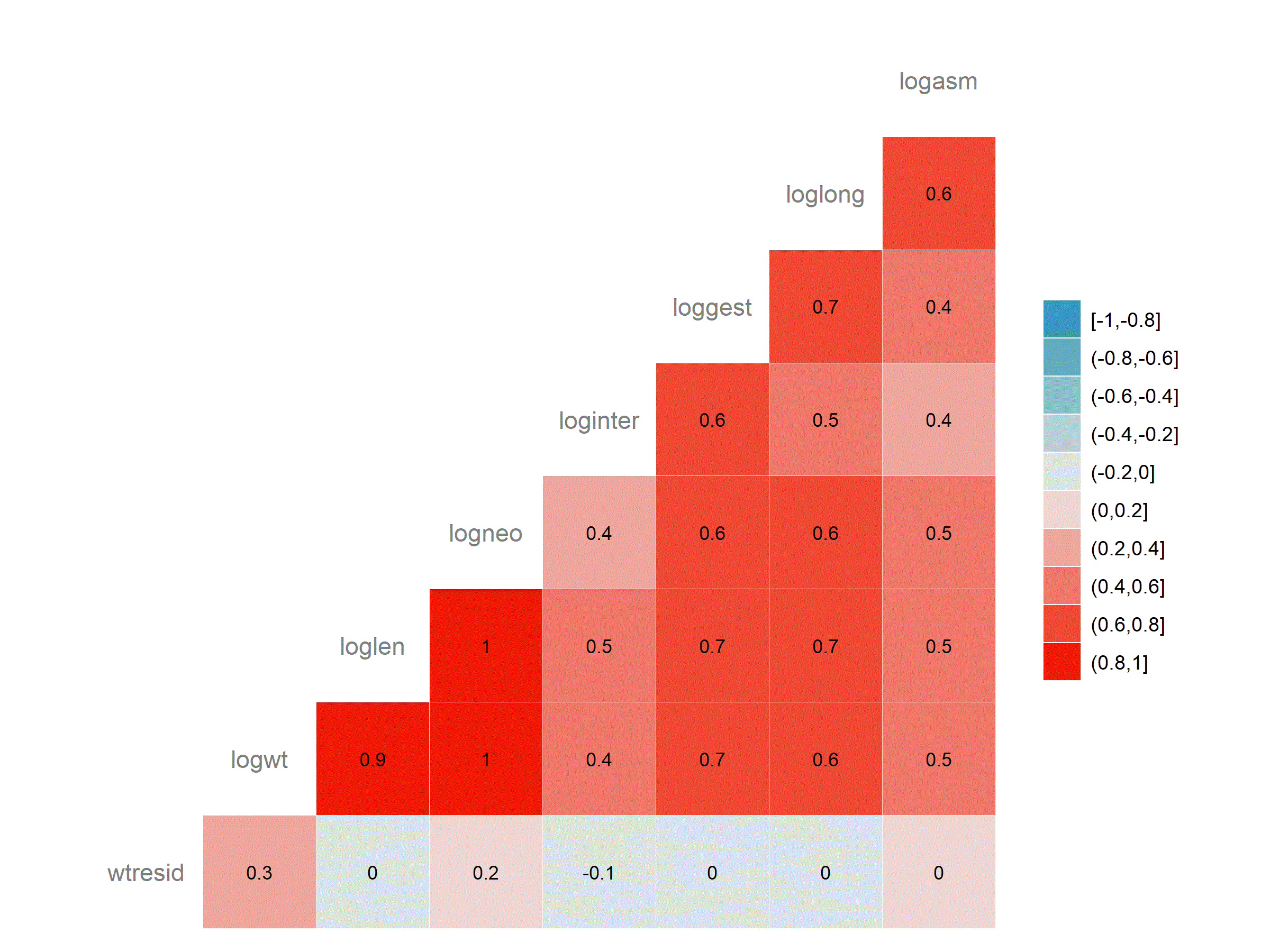


logasm = log10 Age of Sexual Maturity, loglong = log10 Longevity, loggest = log10 Gestation Length, loginter = log10 Interbirth Interval, logneo = log10 Neonate Body Length, loglen = log10 Adult Body Length, logwt = log10 Adult Body Mass, wtresid = Residual of log body mass versus log body length (measure of body shape).
